# The Influence of Event Valence and Emotional States on the Metaphorical Comprehension of Time

**DOI:** 10.3389/fpsyg.2019.00410

**Published:** 2019-03-05

**Authors:** Weiqi Zheng, Ye Liu, Chang Hong Liu, Yu-Hsin Chen, Qian Cui, Xiaolan Fu

**Affiliations:** ^1^State Key Laboratory of Brain and Cognitive Science, Institute of Psychology, Chinese Academy of Sciences, Beijing, China; ^2^Department of Psychology, University of Chinese Academy of Sciences, Beijing, China; ^3^School of Psychology, Beijing Sport University, Beijing, China; ^4^Department of Psychology, Bournemouth University, Dorset, United Kingdom; ^5^Institute of Psychology and Behavior Sciences, Wenzhou University, Wenzhou, China; ^6^College of Psychology, Liaoning Normal University, Dalian, China

**Keywords:** event valence, emotional state, temporal-spatial metaphor, ego-moving, time-moving

## Abstract

Time is generally conceptualized in terms of space as reflected in temporal-spatial metaphors. Two observation perspectives have been proposed in the front-back axis of the temporal-spatial metaphor. One is called “ego-moving perspective” and the other “time-moving perspective.” They are used to represent different relative motion between time and the observer. Previous studies have demonstrated the psychological reality of both perspectives. They also provided evidence that emotion can influence a perspective choice. In general, a positive emotion tends to facilitate the adoption of ego-moving perspective, whereas, a negative emotion tends to promote the adoption of time-moving perspective. However, it is unclear how the motivational dimension of emotion might influence the preference. The current study aimed to address the question by identifying conditions in which emotional valence or motivational attribute affects the choice of time movement perspective. An ambiguous temporal question and a visualized time motion schema were adopted to probe participants' metaphorical representation of time when they were affected by emotion. Study 1 investigated how a future emotional event would affect participants' choice of the time movement perspective. The results showed that positive future events led to a higher propensity to adopt an ego-moving perspective compared with negative future events. Study 2 explored participants' tendency to choose time movement perspective for a vague or neutral future event, after they were induced into a particular emotional state. The results showed that when being in an emotional state of approach-motivation individuals were more likely to adopt an ego-moving perspective. In contrast, being in an emotional state of avoidance-motivation, individuals were more likely to take a time-moving perspective. Taken together, these results suggest that the emotional valence of future events can influence the choice of time movement perspectives; and the motivational dimension of present emotional states plays an important role when contemplating a neutral future event.

## Introduction

Time is one of the most important concepts in our daily life. Without some sense of it, we would be able to do nothing. However, time is an abstract concept that we can hardly see or touch. We learn the concept of space very early and have a strong ability to perceive it. People seem to use mental representation of space to comprehend time unconsciously and automatically (Boroditsky, 2011a). For example, spatial words are routinely used to describe the order and duration of events, such as “forward,” “back,” “long,” and “short” (Traugott, 1978). In linguistics, this phenomenon is called the temporal-spatial metaphor (Clark, 1973). Numerous studies have demonstrated that temporal-spatial metaphors are widely used across different languages and cultures (Radden, 2004; Santiago et al., 2007; Casasanto, 2008). Three spatial axes that are frequently adopted in temporal-spatial metaphors are front-back axis, up-down axis, and left-right axis (Boroditsky, 2001; Chen, 2007; Santiago et al., 2007).

The front-back axis can be used to represent motion in time. There are two kinds of perspectives in the front-back axis of temporal-spatial metaphors. They describe the relative motion between a person and time either as “ego-moving” or “time-moving” (Boroditsky, 2000; Gentner et al., 2002). What the two perspectives have in common is that the future is in front whereas the past is behind the observer. The disparity is that from the “ego-moving” perspective, the “ego,” or the “observer” progresses toward the future (Boroditsky, 2000). For example, “we are creeping up on the deadline” or “I will reach the interview in a few hours.” Conversely, from the “time-moving” perspective, the time is conceived as a river or a conveyor belt on which events are moving from the future to the present (Boroditsky, 2000). For example, “the deadline is creeping up on us” or “the interview will reach me in a few hours.” The psychological reality of the two perspectives of time movement has been demonstrated through several studies (Gentner and Imai, 1992; McGlone and Harding, 1998; Boroditsky, 2000).

### Emotion and Perspectives of Time Movement

Many factors could influence the choice of these perspectives. Our perception of time is intimately connected with our experience of movements in space and spatial reasoning (Boroditsky and Ramscar, 2002; Matlock et al., 2005). Language and culture help us construct fundamental notions of time (Boroditsky, 2011b). Individual differences in personality may also play a role. For example, people who prefer the ego-moving perspective averaged higher degrees of extroversion than people who prefer the time-moving perspective (Duffy and Feist, 2014).

In addition to these factors, emotion also affects our choices of time movement perspective. Emotion is regarded as the foundation of reasoning and thought (Damasio, 1994). From the view of embodied cognition, emotion can create the basis of bodily feeling and spatial environment that help people perceive time and comprehend the temporal-spatial metaphors (Margolies and Crawford, 2008).

Recent studies have revealed that the emotional valence of events can be associated with different perspectives of time movement (e.g., Margolies and Crawford, 2008; Ruscher, 2011; Richmond et al., 2012; Lee and Ji, 2014). A common hypothesis in these studies is that positive emotions are typically associated with the ego-moving perspective, whereas negative emotions are associated with the time-moving perspective. For example, when participants anticipated an enthusiastic event (Margolies and Crawford, 2008), a happy future (Lee and Ji, 2014), or being induced in a happy condition (Richmond et al., 2012), they tended to evoke the ego-moving perspective and reported that they were approaching the event. In contrast, when participants were asked to think of a dreadful future event (Margolies and Crawford, 2008; Lee and Ji, 2014), or in an anxiety/sadness-induced condition (Richmond et al., 2012), they tended to evoke the time-moving perspective and felt that the event was approaching them. Further, Ruscher (2011) has found that after being primed to adopt an ego-moving perspective, participants were more likely to forecast shorter grieving periods in comparison with the time-moving perspective. This demonstrates the influence of temporal-spatial metaphor representation on emotional feelings. Richmond et al. (2012) have also provided bidirectional evidence for the association between an ego-moving perspective and happiness, as well as the evidence for a time-moving perspective being associated with anxiety and depression.

#### The Motivational Dimension of Emotion

The studies summarized above have centered on the relationship between emotional valence and perspectives of time movement. However, not all negative emotions prompt the time-moving perspective. For example, processing an angry event can lead to higher likelihood of choosing the ego-moving perspective (Hauser et al., 2009). Compared with other negative emotions like anxiety and fear, anger can sometimes prompt people to approach and tackle the annoyed situation (Harmon-Jones, 2003). It is known that anger is an emotion that could evoke approaching behavior (Harmon-Jones and Allen, 1998). Although some studies also suggested a link between sadness and approach, it may actually stem from a failure of approach (Carver, 2004). In contrast to sadness, anger stems from the activation of the approach system, which is related to higher levels of left frontal activity (Fox and Davidson, 1988; Harmon-Jones and Allen, 1998). Therefore, the activation of approach motivation is not only associated with positive emotions but also with some negative emotions, such as anger and separation distress (Harmon-Jones et al., 2013). The motivational dimensional model of emotion proposed by Bradley et al. (2001) and Lang (2010) suggests that emotion is rooted in an appetitive motivational system and a defensive motivational system. The nature of approach or avoidance is spatial motion. The arousal of emotion reflects the activation intensity of motivational systems and promotes the appetitive or defensive behavior. The motivational dimensional model of emotion also argues that even if two emotions had the same valence, they could influence the cognitive process differently because of their different orientations of approach and avoidance (Bradley and Lang, 2000; Carver and Harmon-Jones, 2009; Gable and Harmon-Jones, 2010).

This has led to a call to consider motivational direction and valence of emotion as separate dimensions in revealing the role of emotion in temporal-spatial metaphor representation. However, in most previous studies, approach–avoidance and emotional valence were intertwined, where the approach motivation was linked to positive feelings (e.g., happiness, enthusiasm), whereas the avoidance motivation was linked to negative feelings (e.g., anxiety, grief). Although in Hauser et al. (2009)'s study, results showed that compared with neutral emotion, anger as a negative valence but approach-motivated emotion could lead to ego-moving perspective choice. That means the results cannot totally prove whether emotion with the same motivational direction but different valence can lead to a similar perspective. In other words, up to now, there is no consensus as to whether there is a difference between valence dimension and motivation dimension in their influence on the tendency of time movement perspectives. Specifically, it is unknown under which conditions emotional valence contributes more to the choice of a perspective, or in which conditions motivational attribute contributes more to the choice. One purpose of the current study was to address these questions.

#### Future Event Valence and Current Emotional State

When perceiving the relative motion between future events and ourselves, we often face two kinds of situations in daily life. In one case, we know the nature of the event that will happen in the future (e.g., a New Year's party, a hospital appointment, a funeral, etc.), such that our temporal reasoning of the event can be influenced by the emotional valence of the event. In another case, we are not sure whether the nature of the event is good or bad (e.g., a job interview, results of an exam, a new duty, etc.), but we can be in a particular mood when thinking about a future event that may not have a clear valence. In this case, our temporal reasoning of events could be affected by our current emotional state, rather than by the valence of the future event. Although the two situations can also be mixed to various degrees, this study only focused on the unmixed situations.

Whether manipulating future emotional events or inducing emotional feelings, prior research has often focused on emotional events rather than neutral events. Some studies had participants imagine or read scenarios describing future emotional events (Margolies and Crawford, 2008; Hauser et al., 2009), while others had participants write down emotional experience (Lee and Ji, 2014) or watch film clips that induced emotional feelings (Richmond et al., 2012), which could potentially confound the current emotional states and the future event valence. In essence, all the studies did not examine the role of current emotional states on ambiguous or neutral future events.

Thus, in order to distinguish the effects due to valence and motivation dimension of emotion, the current study also aimed to explore how future event valence and current emotional states affect the time movement perspectives on emotional and neutral events.

### The Consistency of the Questions to Distinguish Two Perspectives

A classical method to distinguish time movement perspectives relies on the following ambiguous temporal statement: “The meeting originally scheduled for next Wednesday has been moved forward two days.” To this, participants are asked to answer the question “What day is the meeting now that it has been moved?” (McGlone and Harding, 1998). The phrase “moving forward two days” can be interpreted as moving 2 days closer the future or moving 2 days closer to the present moment. Answering “Friday” implies choosing the ego-moving perspective, because people would imagine themselves moving toward the future, hence “forward” is interpreted as moving in the direction from Wednesday to Friday. In contrast, answering “Monday” means choosing the time-moving perspective, because people would imagine the future moving toward them, hence “forward” is interpreted as moving in the direction that time is moving from Wednesday to Monday (Hendricks and Boroditsky, 2017). In recent studies, this method has been used extensively to investigate how people choose different time movement perspectives (Boroditsky and Ramscar, 2002; Margolies and Crawford, 2008; Hauser et al., 2009).

A potential issue of using a single experimental question is that it might not be generalizable (Duffy, 2014). Richmond et al. (2012) investigated the consistency between participants' responses to the Wednesday's meeting question and other ambiguous questions, such as “a book will be re-edited so that page 10 will move forward 5 pages,” “normally an alarm clock is set for 9 a.m., but the alarm has been moved forward 10 minutes,” and “the winter Olympics normally take place in December but the committee has moved it forward 1 month.” The responses to each question were coded as either ego-moving or time-moving. Results showed consistent responses to different questions hence demonstrate the generalizability of the effect.

In the present study, we further examined the validity of the ambiguous temporal question and the consistency between it and a visualized schema question in a Chinese context. The visualized schema was adapted from Figure 1 of Boroditsky (2000), which is a schematic illustration of the ego-moving and the time-moving perspectives. The ego-moving schema shows a cartoon figure running toward future, whereas, the time-moving schema shows the person sitting still on an ottoman, waiting for the time to approach. The pictures were not questions used in Boroditsky (2000)'s experiment, they were graphical representations. And in our preliminary experiment (to test how long one participant can finish the questionnaire and to examine whether there are some unreasonable aspects in the questionnaire), some participants reflected that they did not clearly understand the meaning of the two pictures. Thus, we added a sentence under each schema to explain what the schema meant. The sentences were taken from Margolies and Crawford (2008). The sentence under the ego-moving schema was “我离[某事件]一天越来越近了(I'm approaching the [event]),” and the sentence under the time-moving schema was “[某事件]这一天离我越来越近了(The [event] is approaching me).” Although there were still sentences in the visualized schema question which might affect participants' selection by language and cultural factors, compared with ambiguous temporal questions these descriptive sentences added under the pictures are straightforward sentences describing two kinds of time movement perspectives, rather than temporal reasoning questions where the relative motion between time and person was not expressed frankly.

### The Present Study

In sum, the goals of the present research were to (1) distinguish the role of motivation and valence dimension of emotion in time movement perception about an emotional or neutral event, and to identify the conditions in which either emotional valence or motivational attribute contributes to the choice of time movement perspective; (2) examine the consistency of the classical ambiguous temporal question with a visualized question and determine whether the classical ambiguous temporal question can successfully distinguish between the two perspectives in a Chinese context. Thus, the highlight of our study was that we considered much more complex situations than previous studies and had higher ecological validity. The present study was the first one to explore the motivation and valence effect of emotion separately on two temporal reasoning conditions and compare their difference: thinking over a particular future event with emotional valence, or a neutral future event with current emotional states.

To achieve these goals, we conducted two studies: In Study 1, we examined whether event valence or motivation dimension played a primary role in time movement perception of emotional future events. In Study 2, we examined whether the valence or motivation dimension of induced mood contributed more to temporal reasoning about neutral future events. To distinguish the role of motivation and valence dimension of emotion, we adopted three kinds of emotion in both studies: happiness, a positive emotion with approach motivation; anger, a negative emotion with approach motivation; and anxiety, a negative emotion with avoidance motivation.

## Study 1

### Method

#### Participants

The participants were 300 students from several universities in Beijing (144 males, 146 females) with an average age of 21.4 (SD = 2.9) years old. All were native Chinese speakers. The participants were randomly assigned to one of three event emotion groups (happy, angry, and anxious), with 100 participants in each. The study was approved by the Ethics Committee of the Institute of Psychology, Chinese Academy of Sciences.

#### Stimuli

The stimuli were three scenarios (see Table 1). Each contained a future event with one of three emotions (happiness, anger, and anxiety) told from the first person's perspective.

**Table 1:** The future events in study 1.

**Emotion**	**Language**	**The stimuli**
Happy	Chinese	家人决定假日带自己去向往已久的地方旅行。
	English	The family decided to take me on a trip during the holiday to the place where I have a long yearning for.
Angry	Chinese	刚在商店买了一双价值不菲的新鞋子,但第二天鞋子质量就出了问题,于是准备去找商家理论。
	English	I noticed there were quality issues with the brand new and very expensive shoes that I bought in the shop yesterday, so I planned to argue with the seller.
Anxious	Chinese	下星期要求上交课程论文,但是我的进度还很落后。
	English	I have to hand in the course paper next week, but my progress is lagging behind schedule.

*The stimuli in this study were presented in Chinese only, the English translation is provided for the convenience of the readers of this article*.

The scenarios were chosen from a pilot study, which had nine scenarios (with three for each emotion). The ratings of the emotional intensity of scenarios were obtained from 20 undergraduate students who did not participate in the later studies. For each scenario, students rated the intensity of each emotion on 9-point Likert scales, where 0 represented no emotion, and eight the strongest emotion). Since the same scenario may elicit different emotions, every scenario was rated on all three emotions (happy, angry, and anxious). The scenarios that scored the strongest mean emotional intensity rating in one emotion, but the lowest in the other two were chosen as the stimuli for this study. For example, the event ultimately chosen for the “happy” group was the one with the highest rating score for happiness but the lowest rating scores for anger and anxiety. Hence the scenarios we selected were the least ambiguous in the sense that they each would selectively elicit one emotion and minimize any influence of the other two.

Two questions were adopted to distinguish time movement perspectives. The first question was a modification of the ambiguous temporal question (McGlone and Harding, 1998), in which the word “meeting” was replaced with the event described in a scenario. For example, “The departure time originally scheduled for next Wednesday has been moved forward two days. What day is the departure day now that it has been moved?” The Chinese translation in the questionnaire is “如果原本出发的一天定于下周三, 但是现在因为某些原因, 要将出发的时间移动两天(既有可能推迟, 也有可能提前), 您认为出发时间被移到了周几?” The reason we chose the Chinese verb “移动” in the question was because it does not contain too much information about the moving direction and is thus spatially and temporally ambiguous. Also, we think the ambiguity should not be decided by the literal meaning of the moving direction in the question, which is easily affected by language and culture. The ambiguity should come from the double choices of the time movement perspectives, which is embodied in the temporal reasoning of “Wednesday's meeting question.” To avoid any confounding factors from the translation, and to remind participants that there are two options they can choose from, we added a detailed explanation after the verb phrase (The meeting might be ahead of schedule; it might also be delayed.) We also balanced the sequence of “提前” and “推迟” in questionnaires. This allowed us to test how the emotional valence of a future event could influence the participant's time motion perception of the event.

The second question was the visualized schema question described earlier in the introduction. The locations of the two perspective schemas in the visualized schema question were counter-balanced throughout the experiment. The ambiguous temporal question was always shown before the visualized schema question. We used a fixed order because the visualized schema question is less ambiguous, which could easily influence the participants' next choice of a time movement perspective, if the more ambiguous question were presented after it. All the questions described in Chinese and their English translations, among all the emotion groups, were listed in Appendix A (Supplementary Materials).

#### Procedure

Firstly, participants were asked to read the scenario and answer the two questions to distinguish time movement perspectives. After the testing questions, they read the scenario again and then rated the emotional intensity of the scenario. The rating was given on a 9-point Likert scale (0–8), where 0 represented no emotional feeling, and eight the strongest emotional feeling. Each scenario was rated for three types of emotions (happiness, anger, and anxiety). The rating task was given after the questions to avoid interference with the question-answering task.

### Results

Firstly, for every group, the participants whose rating scores reflected a correct identification of the emotion associated with the scenario (e.g., in the happy group, they gave happiness a higher score than anger, and anxiety) were included in the final data analysis. The participants who successfully identified the emotion of the events were 90.9% in the happy group, 51.0% in the angry group, 74.0% in the anxious group. All the valid data from Study 1 is available in Appendix B (Supplementary Materials).

Secondly, we used a Chi-square analysis to contrast the percentage of temporal perspectives that participants chose in the two questions and calculated whether the difference among emotions was significant.

**Figure 1:**
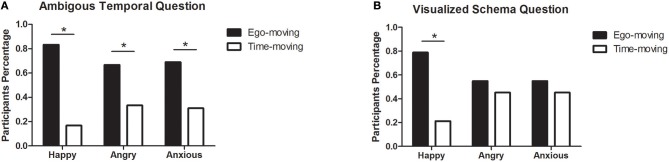
Participants ratio of two perspectives in three emotional groups in Study 1. **(A)** Results of the ambiguous temporal question. **(B)** Results of the visualized schema question. *p < 0.05.

For the ambiguous temporal question, 83.3% of participants in the happy group chose Friday. This indicated their adoption of the ego-moving perspective. The difference between the choice of the two options was significant, χ^2^ (1, 90) = 40, *p* < 0.001. The majority of participants in the angry group (66.7%) and the anxious group (69.0%) also chose the ego-moving perspective rather than the time-moving perspective, χ^2^ (1, 51) = 5.67, *p* = 0.017, and χ^2^ (1, 71) = 10.27, *p* = 0.001, respectively, for the two groups (see Figure 1A).

As for the visualized schema question, 78.9% of participants in the happy group chose the ego-moving perspective. The difference between the two perspective options was significant, χ^2^ (1, 90) = 30, *p* < 0.001. In both the angry and the anxious groups, 54.9% of participants chose the ego-moving perspective. Neither was significantly different from the participants choosing the alternative time-moving perspective, χ^2^ (1, 51) = 0.49, *p* = 0.484, and χ^2^ (1, 71) = 0.69, *p* = 0.406, respectively, for the two groups (see Figure 1B).

Furthermore, for each pair of emotion groups (happy vs. angry, happy vs. anxious, angry vs. anxious), we compared the differences of choosing the two perspectives among all the emotions by chi-square analysis. For both questions, there were significant differences between the happy and angry groups when answering with an ego-moving or a time-moving perspective. The difference between the happy and anxious groups was also significant. More participants chose an ego-moving perspective in the happy group than in the angry and anxious groups. The difference between the angry and anxious groups was not significant (Table 2).

**Table 2:** Pairwise comparisons between emotion conditions within each question in Study 1.

**Pairwise comparisons**	**Ambiguous temporal question**	**Visualized schema question**
Happy-angry	χ^2^ = 5.15^*^	χ^2^ = 8.96^**^
Happy-anxious	χ^2^ = 4.6^*^	χ^2^ = 10.53^**^
Angry-anxious	χ^2^ = 0.08	χ^2^ = 0

* *p < 0.05*,

** *p < 0.01*.

The consistency of the answers from the two questions was also calculated. For each participant, if answers for both questions represented the same time movement perspective, we scored it “1,” otherwise “0.” The values from participants were then summed to measure the whole group's consistency. Therefore, each emotion group's consistency coefficient was the ratio of the whole group's consistency value to the number of participants in the group. The consistency was 0.69 in the happy group, 0.53 in the angry group, and 0.49 in the anxious group.

### Discussion

The results showed that when the ambiguous temporal question was given, most participants in all three groups chose “Friday,” indicating a preference for ego-moving perspective. However, when using the visualized schema question, only those in the happy group clearly favored ego-moving perspective. The results from the both questions also showed a higher consistency between the responses to the two questions in the happy group relative to the angry and anxious groups. This suggests that, compared with a negative-valence emotion, a positive-valence emotion is more likely to trigger a preference for an ego-moving perspective. These results demonstrate that emotional valence, rather than the motivation dimension of a future event, plays a crucial role in modulating the time movement perspective about the event.

Study 1 showed that emotional valence of a future event could affect comprehension of a temporal-spatial metaphor. It is unclear, however, whether emotional valence of the current state can also influence this temporal-spatial metaphor comprehension of a neutral future event. To explore this question, we manipulated the participant's current emotional states prior to the metaphor comprehension tasks in Study 2. Two methods were used to induce a specific emotion into the current state of the participant. In study 2a, stories were used to induce a specific current emotional state, while in study 2b, participants were instructed to recall and write down their own experience related to a specific kind of emotion.

## Study 2a

### Method

#### Participants

The participants were 300 students from several universities in Beijing (166 males, 134 females) with an average age of 21.3 (SD = 2.5) years. All were native Chinese speakers. They were different students from those who participated in Study 1. They were randomly assigned to three emotion groups (happy, angry, anxious), with 100 participants in each group. The study was approved by the Ethics Committee of the Institute of Psychology, Chinese Academy of Sciences.

#### Materials

The stimulus in each participant group was a short story with only 20–30 words that aimed to elicit one of three emotions (happy, angry, and anxious). Hence a total of three stories were used, one per participant group (all three stories are listed in Table 3).

**Table 3:** The stories in Study 2a.

**Emotion**	**Language**	**The stimuli**
Happy	Chinese	暑假的一天,爸爸突然告诉我一个好消息,假期要带我和妈妈一起坐飞机去云南七日游。我喜出望外,高兴地跳了起来。因为我一直向往去这彩云之南的地方看看,欣赏那美丽的自然风光与少数民族独特的风情,一想到放假就能飞去那里,怎能不使人高兴呢？
	English	One day in the summer vacation, my dad suddenly surprised me with good news that he would take our family on a 7-day trip to Yunnan province. I was so happy and jumped with joy. Because I have long yearned for Yunnan and looked forward to seeing the beautiful natural sceneries and to experiencing the unique customs of local Chinese minorities. How can I not get excited when I know I will fly to Yunnan in this vacation?
Angry	Chinese	近日,有网友爆料,在巴黎地铁看到了用中文写的小广告“上门理发”,网友调侃到,小广告终于走出国门,走向了世界。对此,网友“小朱射手座”评论到:“中文小广告都贴到巴黎去了!能不能给国人长点儿脸啊?”网友“小宝1988张”坦言,“小广告漂洋过海,会严重影响中国人的形象”。
	English	Recently, netizens revealed that they noticed adlets with “in-home hair salon” written in Chinese pasted all over the Paris subway. Other netizens joked that Chinese adlets have finally made its way out of the domestic market and reached the international stage. Commenting on this phenomenon, a netizen “Sagittarius Zhu ” exclaimed that “Chinese adlets have now been posted in Paris! It's a shame to all Chinese to witness such disrespectful behavior in a foreign country!” Another netizen “Xiaobao1988zhang” said frankly: “These adlets will seriously damage the image of China.”
Anxious	Chinese	一个毕业生的内心独白:马上就快毕业了,但我对于未来还是很茫然,现实的无奈、梦想的遥远,都让年轻的心倍感压力。看着周围的一些同学对于未来有了很好的规划,对自己也信心满满,我就更加着急了。其中有的同学已经找到了好的工作,有的同学考入了好学校继续深造,还有的同学收到了国外牛校的offer, 再看看自己,工作至今没有着落。
	English	The following excerpt is a monolog from a graduate: I will graduate soon, but I feel confused about my future. My youthful mind is under huge pressure facing the harsh reality and the distant dream. And I feel more worried when I see classmates around me appearing confident with good plans for future. Amongst them, some have already had good job offers, others have been admitted into good universities for further study and a few have obtained offers from top universities abroad. In contrast, I haven't even secured a job.

*The stimuli in this study were only presented in Chinese. The English translation is provided for the convenience of the readers of this article*.

The three stories were selected in advance through a pilot study. Nine stories (three optional stories for each kind of emotion) were rated for their emotional intensity. The raters were 20 university students who did not participate in the later studies. Each story was rated for three emotions (happy, angry, anxious) on 9-point Likert scales. The story with the highest intensity rating in one emotion category but the lowest rating scores in the other two categories was chosen as stimuli.

As in Study 1, two questions were also adopted to distinguish time movement perspectives in Study 2a. The first question was the classical ambiguous temporal question, which was similar to that used in Study 1 except that the event was unrelated to the story. A meeting was used as the future event, hence the question was “The meeting originally scheduled for next Wednesday has been moved forward two days. What day is the meeting now that it has been moved?” The second question was the visualized schema question, which was also similar to that used in Study 1 except that the words of the specific events in the two sentences at the bottom of the two pictures was replaced with “future.” All the questions described in Chinese and their English translations among all the emotion groups are listed in Appendix A.

#### Procedure

Firstly, participants were asked to rate their emotional feelings before reading the story. The same 9-point Likert scale as Study 1 was used for rating the three types of emotions (happy, angry, and anxious). Then they were asked to read the story and answer two questions to distinguish the time movement perspectives.

After that, they were required to read the same story and rate their emotional feelings. The second reading and second rating were required to measure the change in their feelings after reading the story, undisturbed by the process of temporal reasoning.

### Results

According to the difference of the rating scores before and after reading the stories, we decided whether the data would be used for further analyses. The screening standard was that in each emotion group, the rating score of the primed emotion should increase while the two unprimed emotions should stay more or less the same (e.g., in the happy group, only if the rating for happiness increased while the ratings for anger and anxiety changed less, hence we included that data for further analyses). Also, the data inclusion criteria required the rating score of the primed emotion to be larger than unprimed emotions. We used the ratio of the included participants and all the participants to calculate the effective rates in each group, and the results showed that the effective rates of the stories were 70.4% for the happy group, 28.3% for the angry group, and 40.2% for the anxious group. All the valid data of Study 2a is available in Appendix B (Supplementary Materials).

**Figure 2:**
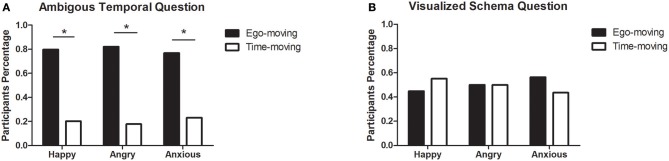
Participants ratio of two perspectives in three emotional groups in study 2a. **(A)** Results of the ambiguous temporal question. **(B)** Results of the visualized schema question. *p < 0.05.

Again, we used a Chi-square analysis to contrast the percentage of the perspectives that participants chose for the two questions and calculated whether the differences among emotions were significant. Results are shown in Figure 2A. For the classical ambiguous temporal question, 79.7% of participants in the happy group chose Friday (representing the ego-moving perspective). The difference between the two options was significant, χ^2^ (1, 69) = 24.36, *p* < 0.001. A similar result was found in the other groups. More participants in the angry group (82.1%) and the anxious group (76.9%) chose the ego-moving perspective, χ^2^ (1, 28) = 11.57, *p* = 0.001, and χ^2^ (1, 39) = 11.31, *p* = 0.001, respectively, for the two groups.

Results for the visualized schema question are shown in Figure 2B. No difference was found between those choosing ego-moving and time-moving perspectives. The percentage of participants choosing the ego-moving perspective was 44.9% in the happy group, χ^2^ (1, 69) = 0.71, *p* = 0.399, 50 % in the angry group, χ^2^ (1, 28) = 0, *p* = 1, and 56.4% in the anxious group, χ^2^ (1, 39) = 0.64, *p* = 0.423.

Results of pairwise comparison of choosing the two perspectives showed no differences among all the emotion conditions (Table 4). The consistency of the answers for the two questions was 0.45 in the happy group, and 0.39 in the angry group, 0.49 in the anxious group.

**Table 4:** Pairwise comparisons between emotion conditions within each question in Study 2a.

**Pairwise comparisons**	**Ambiguous temporal question**	**Visualized schema question**
Happy-Angry	χ^2^ = 0.08	χ^2^ = 0.21
Happy-Anxious	χ^2^ = 0.12	χ^2^ = 1.32
Angry-Anxious	χ^2^ = 0.27	χ^2^ = 0.27

### Discussion

Similarly to Study 1, most of the participants preferred “Friday” as the interpretation of the ambiguous statement contained the temporal-spatial metaphor. This again implies their preference for the ego-moving perspective. However, the result of the visualized schema question in the happy group was quite different. Unlike Study 1, where more participants preferred ego-moving perspective in the happy group relative to angry and anxious groups, the result of the happy group was no different from the other two groups in the current study.

In addition, the pairwise comparison results of choosing the time movement perspective among all the emotion groups showed no significant differences from two questions. Although this may suggest that emotion does not always lead to a modulation of the time movement perspective, it is also possible that the method in this study did not induce a sufficient level of emotion. To explore this possibility, we used a different method to evoke the current emotional states in Study 2b. The method required participants to write down their own experiences with a particular type of emotion.

## Study 2b

### Method

#### Participants

The participants were 102 students from several universities in Beijing (48 males, 88 females) with an average age of 22.6 (SD = 1.7) years. All were native Chinese speakers, who did not participate in Studies 1 and 2a. Participants were randomly assigned to three emotion groups, with 34 in each. Because the method here involved more extensive testing of participants individually in a laboratory setting, we had to reduce the number of participants that we were able to recruit in Studies 1 and 2a, where paper questionnaires were distributed in classrooms. The study was approved by the Ethics Committee of the Institute of Psychology, Chinese Academy of Sciences.

#### Materials and Procedure

After completing necessary demographic information, participants began the task by rating their current emotional states of happiness, anger, and anxiousness on the three 9-point Likert scales. They were then asked to vividly recall and write down their experiences associate with a particular type of emotion, depending on the emotion group they were assigned. They were given 20 min to write down their experiences in detail. They were encouraged to use the full duration to immerse themselves in their past emotional experience. After this, participants were asked to rate their feelings again on the same three scales. Once rating was complete, the participants were required to answer the same two questions as in Study 2a. All the questions described in Chinese and their English translations among all the emotion groups are listed in Appendix A.

### Results

Data were included for further analysis if the rating scores for the induced emotion were higher after an emotional experience of the past was recalled and written down. This screening criterion was the same as in Study 2a. The results show that 97.1% of the participants in the happy group, 76.5% in the angry group, and 44.1% in the anxious group produced a higher rating of the induced emotion. All the valid data of Study 2b is available in Appendix B (Supplementary Materials).

**Figure 3:**
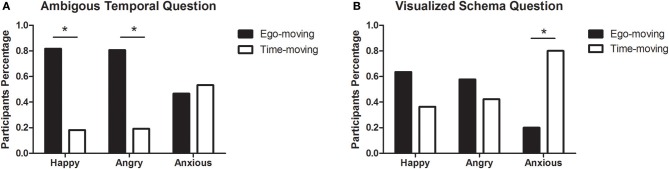
Participants ratio of two perspectives in three emotional groups in study 2b. **(A)** Results of the ambiguous temporal question. **(B)** Results of the visualized schema question. *p < 0.05.

For the classic ambiguous temporal question, significantly more participants (81.8%) in the happy emotion group chose Friday as the answer, indicating their adoption of the ego-moving perspective, χ^2^ (1, 33) = 13.36, *p* < 0.001. The same result was also found in the angry emotion group, where 80.8% of participants chose the ego-moving perspective, χ^2^ (1, 26) = 9.85, *p* = 0.002. In the anxious emotion group, however, only 46.7% of participants chose the ego-moving perspective. This was not significantly different from those who chose the time-moving perspective, χ^2^ (1, 15) = 0.07, *p* = 0.8 (see Figure 3A).

As for the visualized schema question, 63.6% of participants in the happy emotion group chose the ego-moving perspective, which was no more than those who chose the time-moving perspective, χ^2^ (1, 33) = 2.46, *p* = 0.117. A similar, non-significant result was found in the angry emotion group, where 57.7% of participants chose the ego-moving perspective, χ^2^ (1, 26) = 0.62, *p* = 0.433. In contrast, 80% of participants in the anxious emotion group chose the time-moving perspective, significantly more than those who chose the time-moving perspective, χ^2^ (1, 15) = 5.4, *p* = 0.02 (see Figure 3B).

Results of pairwise comparisons of choosing the two perspectives among all the emotions are shown in Table 5. For both questions, there was no difference between happy and angry groups, while the differences between happy and anxious groups, and angry and anxious groups were significant. The consistency of the answers for the two questions was 0.64 in the happy group, and 0.46 in the angry group, 0.6 in the anxious group.

**Table 5:** Pairwise comparisons between emotion conditions within each question in Study 2b.

**Pairwise comparisons**	**Ambiguous temporal question**	**Visualized schema question**
Happy-angry	χ^2^ = 0.01	χ^2^ = 0.22
Happy-anxious	χ^2^ = 6.17^*^	χ^2^ = 7.86^**^
Angry-anxious	χ^2^ = 5.11^*^	χ^2^ = 5.49^*^

* *p < 0.05*,

** *p < 0.01*.

### Discussion

The results of the ambiguous temporal question showed no difference between choosing “Friday” (ego-moving perspective) and “Monday” (time-moving perspective) in the anxious group, although most participants in the happy and angry groups preferred “Friday.” However, the results of the visualized schema question showed no more preference for the ego-moving perspective in the happy and angry group, although most participants in the anxious group adopted the time-moving perspective.

Participants in the happy and angry groups produced similar responses to the two questions, while the anxious group produced different results from the happy and the angry groups. The results suggest that, compared with an approach-motivation emotional state (happy or angry), participants in avoidance-motivation emotional states (anxious) were more likely to take time-moving perspective.

Results here demonstrate that approach-avoidance motivation of the current emotional state, rather than the valence of the emotional state, plays a crucial role in perspective choice of time movement when people comprehend the temporal-spatial metaphor of a neutral future event.

## General Discussion

The current study adopted an ambiguous temporal question and a visualized time motion schema to probe people's metaphorical representation of time when they were affected by emotion. We first investigated how the emotional value of a future event could affect people's choice of time movement perspective. The results showed that positive events could lead to a higher propensity to adopt an ego-moving perspective compared with negative events. We next explored how a particular emotional state could affect time movement perspective on a neutral future event. The results showed that participants in approach-motivation emotional states were more likely to take an ego-moving perspective, whereas those in avoidance-motivation emotional states tended to take a time-moving perspective.

### The Ambiguous Question in Chinese Context

Participants in both of our studies were more likely to choose the ego-moving perspective over the time-moving perspective, when they were facing the classical ambiguous temporal question. This is quite different from the results of prior research with English speakers. In Rothe-Wulf et al. (2015), where the English version of the same question was used without any manipulation, roughly half of the native speakers interpreted “forward” as toward the future, and half interpreted it as toward the past. Hence a reasonable explanation for the inconsistent findings could be that the ambiguity of the classical question was weakened after it was translated into Chinese. Indeed, some cross-linguistic investigations of the classical ambiguous temporal question have shown that the ambiguity of the question is not universal. For instance, when the question was translated into German (using the term *vorverlegen* for “moved forward”), the vast majority of participants chose “Monday” (Beller et al., 2005). This demonstrates that the German translation altered the ambiguous nature of the English version. More recently, Rothe-Wulf et al. (2015) found that, unlike English speakers, German and Swedish speakers were not susceptible to spatial priming when the question was translated into their own languages. Our results further corroborate these observations.

Another surprising finding in our study was that Chinese speakers had a tendency to adopt the ego-moving perspective from the ambiguous temporal question, which appeared contrasting with some previous studies that were also tested in Chinese speakers (Lai and Boroditsky, 2013; Chen, 2014). In Chen (2014)'s study, the conclusion that the “time-moving perspective” was more commonly used in Chinese was mainly from corpus analysis. And in Lai and Boroditsky (2013)'s study, ambiguous meeting question and clock question were used to test Mandarin, English, and Bilinguals' perspective tendency. Both questions showed that English speakers are indeed more likely to take an ego-moving perspective than Mandarin speakers. However, we could not draw a conclusion that our findings have totally overturned previous findings. The first possible reason is that the translation of “moving forward” in the “ambiguous meeting question” in our study is “移动”, while in Lai and Boroditsky (2013)'s study, the verb phrase translation is “往前挪” which contains a direction. Therefore, the difference between the two kinds of translations might contribute to the testing results. Also we found that the results of the visualized schema question in our study do not show a stable tendency of “time-moving perspective” like the ambiguous meeting question. Therefore, the tendency reflected from the ambiguous meeting question in our study should still be questioned.

Furthermore, we can see that the consistency value between the two questions in some emotion groups is not high in both of our studies. The reason might be that the two questions reflected different concepts. The visualized schema question describes the spatial relationship between people and event more straightforwardly because the schema contains the spatial image and the temporal sentence at the same time; while the ambiguous temporal question is more like a simple temporal reasoning question than the visualized schema because the literal information is only about the time sequence, so participants need to imagine the motion direction of the event in their mind. Although the concept of space helps us conceptualize the concept of time, there are notable distinctions between the two concepts. A core feature of time is transience, which is not exhibited by space (Evans, 2013). Thus, time cannot be captured by metaphors that do not make use of the transient aspect. The unique character as a fundamental and inalienable feature of our experience will ultimately resist our attempts to explain time in terms of anything else (Galton, 2011; Duffy and Evans, 2017). This character can help to explain why the consistency between the two questions is not very high.

### The Emotional Valence of Future Events

In line with previous studies (Margolies and Crawford, 2008; Ruscher, 2011), we found a general tendency that future events with positive valence led to a preference for the ego-moving perspective, compared with negative valence events. This is not hard to comprehend because looking forward to positive events and keeping away from negative ones is consistent with the human nature of seeking pleasure and avoiding pain.

However, in Study 1, the answers from the visualized schema question showed asymmetry between the two perspectives. Participants tended to adopt the ego-moving perspective when interpreting happy events, but their choice between the two different perspectives was not affected when interpreting the angry and anxious events. A possible explanation is that people are more familiar with forwarding motion when approaching the future events. That is, forward motion is deeply entrenched in everyday locomotion, whereas, people are far less familiar with backward motion (Matlock et al., 2011; Duffy and Feist, 2017). Therefore, a prior preference to ego-moving perspective, combined with the facilitation from happy events, increases the likelihood of choosing the ego-moving perspective. In contrast, angry and anxious events make people stiff which can promote people to think time is moving toward them, and the effect will counteract the prior preference to ego-moving perspective. Thus, there is no significant difference between the two choices among all the participants.

### The Motivational Dimension of Current Emotional States

Results of the visualized schema question in Study 2b showed that participants tended to take the time-moving perspective when they were in an anxious mood. When they were in a happy or angry mood, however, the preference for ego-moving perspective was not higher than for time-moving perspective. This result was drastically different from that in the anxious group where most of the participants chose time-moving perspective. The results seemed to contradict with Study 1, where the result of the angry group was similar to the anxious group, but different from the happy group. The reason for the difference might be that anger not only represents the negative emotion but also correlates with the approach motivation to some degrees. The weight of the function of negative valence or approach-motivation dimension might be different in various conditions.

Although anger often leads to an approach motivation, there is evidence to suggest that the motivational direction of anger is uncertain. For example, active soccer players in an avoidance condition showed an activation of the left frontal region, a brain area that was known to be associated with angry emotions (Wacker et al., 2003). Hence the study implied that anger is also related to avoidance motivation in some situations. The connection between anger and motivation is processed during the expression period of anger. According to Zinner et al. (2008), “Anger out” is to express the feeling in an explicit way. This is related to approach motivation. In contrast, “anger in” is to depress the feeling. This is related to avoidance motivation. In our Study 2b, the results showed that participants in an angry state were more likely to choose the ego-moving perspective. Hence, we speculate that participants expressed “anger out” accompanied with approach motivation.

Furthermore, Study 2a found no difference between choosing the two perspectives options in all groups, while more participants in Study 2b tended to take the time-moving perspective when they were in anxious mood. The difference might arise from the emotion evoking method. In Study 2a, we used a short story to evoke participants' emotional feelings. The manipulation might not strong enough to influence a sufficient level of emotion. In Study 2b, we asked participants to write down their experiences. It may have created much stronger emotion, which could have influenced the reasoning about the temporal representation in a spatial relationship.

Most previous work has focused on the effect of emotional valence on cognition. Our study is the first attempt to examine the effect of the motivational dimension of current emotional states on the time movement perspective reasoning of a neutral event. The motivational direction involves potential spatial motion, hence emotional states with motivational directions should provide spatial property for time movement cognition and influence people's perception of time. That is, happiness and anger provide approach motion to contribute to the ego-moving perspective, while anxiety provides avoidance motion to contribute to the time-moving perspective.

Overall, from the results of the two studies, we can find that emotion will affect the temporal-spatial metaphor reasoning in a complicated way, and different dimensions of emotion will dominate the judgment and choice in different situations. The findings were more aligned to the theory of appraisal-tendency framework (ATF; Lerner and Keltner, 2000; Lerner and Tiedens, 2006) to some degree. The ATF emphasizes that a range of cognitive dimensions (including, but not limited to valence) differentiates emotional experience and effects. It also proposes that emotions are associated with people's cognitive appraisals of a situation, such that each emotion is defined by a specific pattern of cognitive appraisals. Therefore, the present study also contributed to the construction of ATF from the point of temporal reasoning in life.

### Limitations and Prospects

In this study, we have examined the influence of the objective event valence and the current subjective emotional state on temporal-spatial metaphor comprehension. However, as Gilbert and Wilson (2007) pointed out, our hedonic reaction to a future event is determined by the combination of the future event valence and other contextual factors (such as our current thoughts and present bodily states). This means the objective event valence can also influence our mental simulation for the future event. Therefore, when we reason about the effect of an emotional future event on the comprehension of the temporal-spatial metaphor in Study 1, there is no guarantee that all the participants are in a neutral mood. It is also difficult to guarantee that the participants in Study 2 had no emotional preference for the neutral event set by experimenters.

Although the current study has attempted to analyze the effect of the event valence and emotional state separately, the two factors are likely to entangle in reality. Therefore, future research will need to explore how the interaction of the event valence and participant's current emotional state affects the comprehension of temporal-spatial metaphors.

On the other hand, participants in our study were not distinguished by their characteristics. Thus, future research may explore the intermediation of participants' sensitivity of trait behavioral approach or avoidance. For example, we could use the BIS/BAS Scales to run separate analyses according to participants' BAS and BIS sensitivity (Carver and White, 1994). Also, we should use standardized tests to measure participant's emotions to exclude the influence of alexithymia (people who have trouble identifying and describing emotions and who tend to minimize emotional experience and focus attention externally) by using TAS-20 (Yi et al., 2003).

Recently, a new paradigm using 2D or 3D space to allocate valenced concepts was adopted to study valence-space metaphors (Marmolejo-Ramos et al., 2017, 2018). The 3D results indicated that positive concepts were placed in high locations and near the participants' position, and negative concepts were placed in low locations and far from the participants' position, while neutral concepts were placed in between positive and negative concepts. As for our study, a similar approach could be used to test the space allocation of positive and negative events (for Study 1), or neutral events processed with valenced mood by participants (for Study 2). The allocation task could be conducted after the time movement perspective choosing task. In this way, we can test the valence and motivational dimension of those events on the psychological level through valence-space metaphors, which might further verify our current findings.

## Conclusion

The metaphorical representation of time movement is not only shaped by spatial experiences, but also influenced by the emotional valence of events and subjective emotional states. The results suggest that the emotional valence of events dominates the adoption of time movement perspectives when perceiving the event itself; and the motivational dimension of current emotional states plays the primary role when reasoning about a neutral future event. Emotion will affect the temporal-spatial metaphor reasoning in a cognitive appraisal way.

## Data Availability

All datasets generated for this study are included in the manuscript and/or the supplementary files.

## Author Contributions

WZ, YL, and XF contributed in designing the experiment, analyzing the data, and writing the manuscript. WZ contributed in collecting the data, and CL, Y-HC, and QC contributed in writing the manuscript.

### Conflict of Interest Statement

The authors declare that the research was conducted in the absence of any commercial or financial relationships that could be construed as a potential conflict of interest.
