# Supplementary material for: The Influence of Event Valence and Emotional States on the Metaphorical Comprehension of Time
Source: Front Psychol. 2019 Mar 5;10:410. doi: 10.3389/fpsyg.2019.00410 (PMC6411794; doi:10.3389/fpsyg.2019.00410)
Supplement: Supplementary file 2 [file Data_Sheet_1.PDF]

# Appendix A

**Study 1 Question 1 (the ambiguous temporal question) & Question 2 (the visualized schema question)**

**Happy emotion group**

Question 1: 如果原本出发的一天定于下周三，但是现在因为某些原因，要将出发的时间移动两天（既有可能提前，也有可能推迟），您认为出发时间被移到了周几？（ ）

A、下周一　　　B、下周五

English translation: The departure time originally scheduled for next Wednesday has been moved forward two days for some reason. What day is the departure day now that it has been moved?

A、Next Monday　　　B、Next Friday

Question 2: 请比较下面两张图片及文字说明，您认为哪张图片及其下方的文字说明更符合您此时的心情？（ ）

Please compare the following two pictures and the literal statements, which one do you think is closer to your feeling?

A、
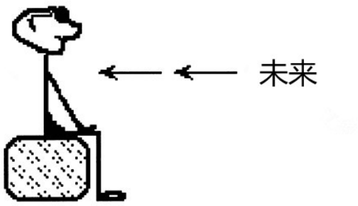


（出发这一天离我越来越近了。）

(The departure day is approaching me.)

B、
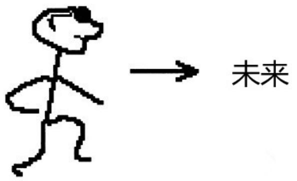


（我离出发这一天越来越近了。）

(I'm approaching the departure day.)

*Note. "未来" means "future"*

**Angry emotion group**

Question 1: 如果原本与商店约定下周三协商这个问题，但是现在因为下周三商店要进行周年庆，所以要将协商的时间移动两天（既有可能提前，也有可能推迟），您认为预约的协商时间被移到了周几？（ ）

A、下周一　　　B、下周五

English translation: The negotiation with the store originally scheduled for next Wednesday has been moved forward two days for the anniversary celebration of the store that day. What day is the negotiation time now that it has been moved?

A、Next Monday　　　　B、Next Friday

Question 2: 请比较下面两张图片及文字说明，您认为哪张图片及其下方的文字说明更符合您此时的心情？（ ）

Please compare the following two pictures and the literal statements, which one do you think is closer to your feeling?

A、

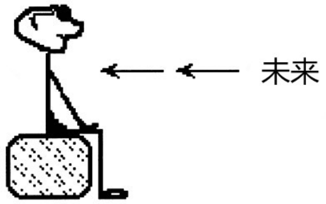


（预约协商这一天离我越来越近了。）

(The negotiation day is approaching me.)

B、

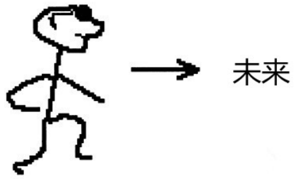


（我离预约协商这一天越来越近了。）

(I'm approaching the negotiation day.)

## Anxious emotion group

Question 1: 如果原本老师定于下周三收论文，但是现在因为某些原因，老师要将收论文的时间移动两天（既有可能提前，也有可能推迟），您认为老师将时间移到了周几？（　）

A、下周一　　　　B、下周五

English translation: The paper due originally scheduled for next Wednesday has been moved forward two days for some reason by the teacher. What day is the paper due now that it has been moved by the teacher?

A、Next Monday　　　　B、Next Friday

Question 2: 请比较下面两张图片及文字说明，您认为哪张图片及其下方的文字说明更符合您此时的心情？（ ）

Please compare the following two pictures and the literal statements, which one do you think is closer to your feeling?



This is a provisional file, not the final typeset article

A、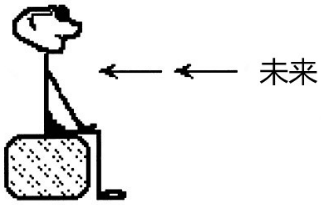


B、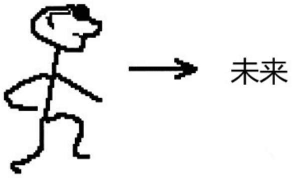


（交论文这一天离我越来越近了。） （我离交论文这一天越来越近了。）

(The paper due day is approaching me.) (I’m approaching the paper due day.)

**Study 2 Question 1 (the ambiguous temporal question) & Question 2 (the visualized schema question)**

Question 1: 假设您所在的学院原定于下周三要给全院同学召开一个会议，但是现在因为某些原因，要将会议时间移动两天（可能提前，也可能推迟），您认为会议的时间被移到了周几（ ）

A、下周一 B、下周五

English translation: The meeting holding by the college for all students originally scheduled for next Wednesday has been moved forward two days for some reason. What day is the meeting now that it has been moved?

A、Next Monday B、Next Friday

Question 2: 请比较下面两张图片及文字说明，您认为哪张图片及其下方的文字说明更符合您此时的心情？（ ）

Please compare the following two pictures and the literal statements, which one do you think is closer to your feeling?

A、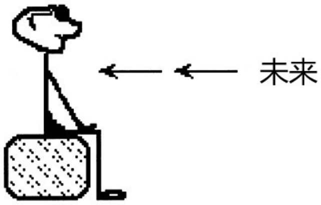


B、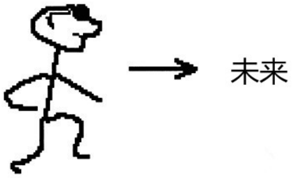


（开会这天离我越来越近了。） （我离开会这天越来越近了。）

(The meeting is approaching me.) (I’m approaching the meeting.)

*Note. Both question 1 and 2 are the same in all emotional states groups in Study 2a & 2b, because Study 2 aimed to explore the temporal reasoning for neutral and vague future events.*
